# Humic Lake Exhibits Higher Microbial Functional Gene Diversity and Weaker Gene Interaction Efficiency than a Common Alkaline Lake

**DOI:** 10.3390/biology11101448

**Published:** 2022-10-01

**Authors:** Dan He, Yuanyuan Liu, Qinglong Wu, Yuyang Peng, Lijuan Ren

**Affiliations:** 1 Guangdong Provincial Key Laboratory of Microbial Culture Collection and Application, State Key Laboratory of Applied Microbiology Southern China, Institute of Microbiology, Guangdong Academy of Sciences, Guangdong Detection Center of Microbiology, Guangzhou 510075, China; 2 State Key Laboratory of Lake Science and Environment, Nanjing Institute of Geography and Limnology, Chinese Academy of Sciences, Nanjing 210008, China; 3 Department of Ecology, Institute of Hydrobiology, Jinan University, Guangzhou 510632, China; 4 Sino-Danish Centre for Education and Research, University of Chinese Academy of Sciences, Beijing 100049, China; 5 Center for Evolution and Conservation Biology, Southern Marine Sciences and Engineering Guangdong Laboratory (Guangzhou), Guangzhou 511458, China

**Keywords:** humic lake, microbial community, functional gene, nutrient cycling

## Abstract

**Simple Summary:**

The humic lake represents a special kind of aquatic ecosystem with high humic substances, low irradiance, and a high potential for greenhouse gas emissions. Despite the special environment and biogeochemical processes in humic lake water, knowledge about the underlying microbial-driven functions remains elusive. Here, we studied the compositions and functional gene structures of microbial communities in a humic lake (HL) and a reference weakly alkaline lake (RAL). We found that the high organic matter content in the HL supported higher gene diversity; and, specifically, the carbon and nitrogen fixations, the degradation of various types of carbon, methane oxidation and methanogenesis, ammonification, denitrification, and assimilatory N reduction might be enhanced more in the HL than in the RAL. By contrast, the humic fractions in the HL might reduce microbial metabolic potential for sulfur oxidation and phosphorus degradation. The potential interactions between different functional microorganisms might be down-regulated provided that there were more easily acquired nutrients in the HL. Overall, our results showed the functional gene “landscape” of microbial communities in the surface water of a humic lake, which helps understand the biogeochemical processes and the remediation of organic matter pollution in lacustrine ecosystems.

**Abstract:**

Humic lakes (HLs) are special water bodies (high organic matter content, low pH, and low transparency) that are important sources of major greenhouse gases. The knowledge about microbial functional potentials and the interactions among different genes in HL water has been scarcely understood. In this study, we used 16S rRNA gene sequencing and the GeoChip 5.0 to investigate microbial community compositions and functional gene structures in an HL and a reference weakly alkaline lake (RAL). The HL microbial communities showed distinct compositions and functional gene structures than those in the RAL. The functional gene diversity was significantly higher in the HL than in the RAL. Specifically, higher gene relative intensities in carbon and nitrogen fixations, the degradation of various types of carbon, methane oxidation and methanogenesis, ammonification, denitrification, and assimilatory N reduction were observed in the HL samples. By contrast, the metabolic potentials of microorganisms involved in dissimilatory N reduction, phosphorus degradation, and sulfur oxidation were weaker in the HL than in the RAL. Despite higher functional gene diversity, the interaction efficiency among genes (reflected by network geodesic distance and clustering coefficient) might be reduced in the HL. Different functional microbes may develop less interdependent relationships in acquiring nutrients given the high resource availability in the HL. Overall, the enhanced microbial metabolic potentials and less efficient functional interactions might have great consequences on nutrient cycling and greenhouse gas emissions in the HL ecosystem.

## 1. Introduction

The humic lake (HL) is a distinct, dark-colored lake, with poor light transparency, low pH, low oxygen, and high content of humic substances (HSs), usually due to a large input of allochthonous (terrestrially derived) organic matter [1,2]. HSs are complex and heterogeneous mixtures formed by biochemical and chemical reactions during the decay and transformation of biomass, a process known as humification [3]. They represent one of the most important components of the total carbon on earth and comprise 50–75% of the dissolved organic carbon (DOC) in water [4]. The high concentration of HLs in the lake had a great role in the carbon and other nutrients’ cycling processes [5], which were largely driven by planktonic microorganisms [1,6].

Due to the general absence of planktivorous and piscivorous fish, the trophic interactions between microbes and other organisms might be less complex in the HL ecosystem [7]. The specialized physicochemical and biological conditions have possibly resulted in distinct microbial communities and their functions. The pelagic microorganisms contributed greatly to the primary and secondary production in the HLs [8], since the enriched organic carbon promoted microbial growth and the dark humic substances hindered the growth and production of aquatic plants [1]. Evidence has shown that the unique environmental conditions of HLs can result in unique bacterial populations, such as the soil II-III clade of Actinobacteria [9]. The specialized bacterial populations may conduct differential functions in HLs than in common lakes. There has been emergent evidence about microbial community compositions in HLs [9,10] and the functions of some specific taxa in the degradation of organics in HLs [11,12].

Microbial functional gene compositions provide more direct evidence than taxonomic compositions in inferring the material cycling and functional processes in an ecosystem [13]. Among technologies used to study microbial functional genes, the GeoChip is a high-throughput microarray-based genomic technology to study various biogeochemical processes and functional activities [14]. There has been some works using GeoChip to study microbial functional genes in natural lakes and artificial aquatic ecosystems. The carbon, nitrogen, phosphorous, and sulfide cycling functions and the metabolic pathways have been investigated [15,16,17]. Gene compositions and functional potentials can be regulated by environmental variables such as pH, DOC, and nitrogen nutrients [18,19,20]. For HL lakes, the specific functions such as the degradation of recalcitrant carbon, the utilization of glycolate [21], and the transformation of organic pollutants [22] have already been studied. These functions could show elevated activity in HLs and have temporal patterns linked with other organisms in water. However, little is understood about the whole-community functional genes and the potential interactions of symbiotic genes in HLs.

Here, we studied a HL (pH 5.16) and a reference common lake (weakly alkaline, pH 8.3, RAL) in Denmark. The GeoChip 5.0 was used to investigate the functional gene differences in the two lakes. The gene association networks were used to infer the potential interactions among different symbiotic functional genes. We hypothesized that (a) the functional gene composition and their functional activities were greatly different between the HL and the common RAL, and (b) the special physicochemical traits in the HL may result in disparate interaction patterns of functional genes than those in the reference common lake. Our results may provide clues for understanding element cycling processes and the amendment of organic matter pollution in lacustrine ecosystems.

## 2. Materials and Methods

### 2.1. Sampling and Water Properties Determination

We selected two lakes with significantly different total organic carbon (TOC) contents. One of the lakes had a TOC value of 31.90 mg·L^−1^ and a pH value of 5.16, hereafter referred to as HL. The TOC content of the other lake was 4.01 mg·L^−1^ (pH, 8.30), hereinafter referred to as RAL. According to the shape and size of the lake, six points with a distance of no less than 10 m from the border were randomly chosen for the sampling. They were uniformly distributed across the whole lake to best represent water samples from the lake.

About 500 mL of surface water (0–50 cm) was gathered with an aseptic plastic bottle at each sampling site. The water samples were filtered through 0.2-μm-pore-size Isopore filters (Merck Millipore, Billerica, MA, USA), and the filters were stored at −20 °C for further analysis. The water conductivity and pH were measured in situ with the YSI650 MDS multiport at each sampling site. About 300 mL of surface water was simultaneously sampled for determinations of the contents of total organic carbon (TOC), total nitrogen (TN), total phosphorus (TP), total iron (TFe), and nitrate/nitrite nitrogen (NO_x_^−^) [23].

### 2.2. DNA Extraction and GeoChip Analysis

A phenol–chloroform extraction method [24] was used to extract the microbial DNA from the stored filters and purified using the Wizard DNA clean-up kit (Promega, Madison, WI, USA). The purified DNA was then quantified using the PicoGreen dsDNA Assay kit (Invitrogen, Waltham, MA, USA). Finally, the DNA samples were put on the GeoChip 5.0 platform for analyzing different functional genes. The sample preparation and analysis steps were described in the work [14].

### 2.3. Data Preprocessing

The raw data from the GeoChip platform were uploaded to the web-based pipeline (http://ieg.ou.edu/microarray/, accessed on 1 October 2021) for analysis [25]. Briefly, the following procedures were taken: (a) signals with a signal/noise ratio smaller than 2 (poor quality or noise signals) were discarded before downstream analyses; (b) to reduce the biased effects caused by too low and too high gene intensities, intensities greater than 1 were taken the logarithms and divided by the mean signal intensity; (c) the relative signal intensity (as a proxy for metabolic potential) was finally generated by standardizing the signal values based on the total probe number of each sample [26].

### 2.4. Network Construction

For both lakes, the *cellobiase*, *FTHFS*, *gdh*, *rubisco*, *glucoamylase*, *mcrA*, *narG*, *nrfA*, *sox*, and *xylA* (descriptions of the functional gene groups and their coded enzymes are shown in Table S1) were the main gene groups involved in element cycling. They were selected to construct a molecular ecological network based on the correlations between different genes. The random matrix theory (RMT) approach was used to determine the correlation threshold for non-random associations from the correlation matrix [27,28]. The standardized Geochip data were uploaded to a web-based pipeline for network analysis (http://ieg4.ou.edu/MENA/, accessed on 1 January 2022) [29]. In the pipeline, the data preprocessing, correlation calculation, and the RMT-based determination of the adjacency matrix were done in sequence. Finally, the functional gene network was constructed from the adjacency matrix. The network topology was visualized by Cytoscape 3.8.0. [30]. For simplicity, we chose the top five genes with high connectivity to display the important network structure.

### 2.5. Statistical Analyses

The alpha diversity index and phylogenetic information for functional genes in all samples were calculated in the web-based pipeline (http://ieg.ou.edu/microarray/, acessed on 1 February 2022). The differences in alpha diversity index and gene relative intensities between the two lakes were tested for significance by the Student *t* tests. Three non-parametric multivariate (Adonis, Anosim, and MRPP) statistical methods were used to test the differences in gene compositions between the two lakes. The above statistical tests were done in the R language environment with the packages “vegan” [31] and “base” [32].

## 3. Results

### 3.1. Environmental Characteristics

There were distinct differences in the physical and chemical properties between the HL and the RAL (Table 1). The RAL was much larger in area than the HL. The HL was an acidic lake with a pH of 5.16, much lower than the pH of the RAL sample (8.30). There were more nutrients in the water in the HL than in the RAL. For example, the total organic carbon (TOC) was about seven-fold higher in the HL than in the RAL. The contents of total nitrogen, total phosphorus, and total ferrous in the HL were about 2–4 times those in the RAL. The content of dissolved inorganic-N (NO_x_^−^) was also slightly higher in the HL water. The water conductivity was similar in the two lakes.

### 3.2. Overview of Microbial Functional Gene Composition and Structure

**Figure 1:**
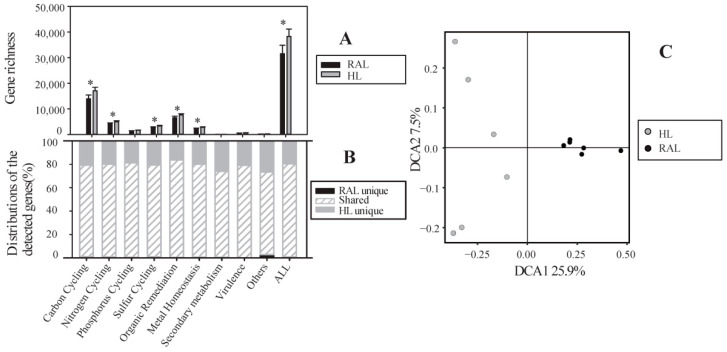
Differentiations between the HL and the RAL in microbial functional genes. (**A**) gene richness, significant differences between the HL and the RAL were signified with the “*” above the middle of bars for different gene categories; (**B**) the distributions (shared or unique) of the detected genes in certain biogeochemical cycling processes; and (**C**) the plot showing detrended correspondence analysis results based on the Bray–Curtis dissimilarity of functional gene compositions.

A total of 38,988 genes were detected from all samples, grouped altogether into 12 functional gene categories (e.g., carbon, nitrogen, phosphorus, and sulfur cycling, organic metabolism, metal homeostasis, secondary metabolism, virulence, and others). Phylogenetically, 1065 genes came from archaea, 3708 genes from fungi, 33,732 genes from bacteria, and 483 genes were unclassified. The HL exhibited significantly higher gene richness (mean ± SD, 32,463 ± 3158) than the RAL (26,488 ± 3596) (Student *t* test, *p* < 0.05, Figure 1A).

**Figure 2:**
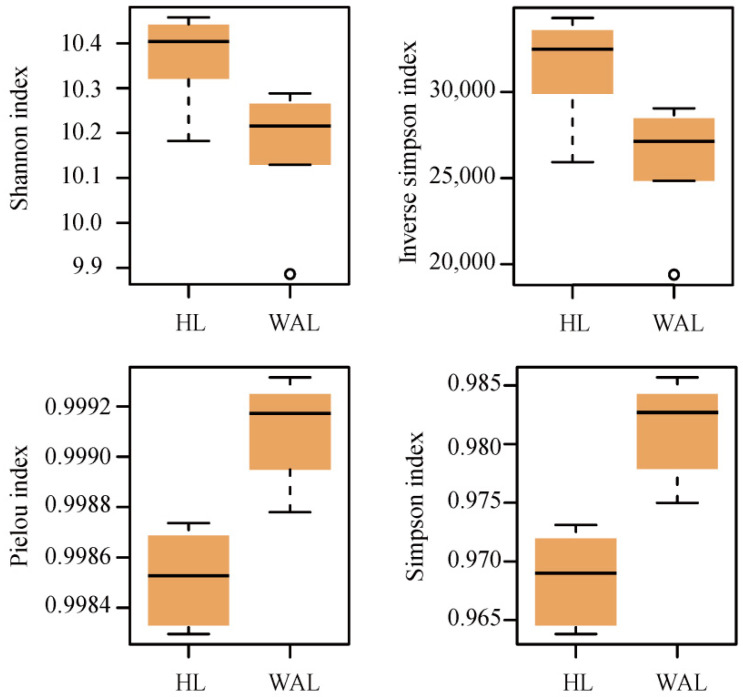
The alpha diversity of microbial functional genes in the HL and the RAL. Four alpha diversity indexes were used, including the diversity indexes (the Shannon-Weaver index and the inverse of Simpson) and the evenness indexes (the Pielou’s evenness and the Simpson’s evenness).

The two lakes shared nearly 79% of the total genes, and the HL had many more unique genes (number: 7430) than the RAL (number: 697). Among the unique genes in HL samples, the proportion of secondary metabolism genes was the highest (Figure 1B). Like the situation for gene number, the HL had significantly higher diversity (Shannon index and Inverse Simpson index) but lower evenness (Pielou’s and Simpson’s evenness) values than the RAL (Figure 2). Overall, significant differences in microbial functional gene compositions were observed between the HL and RAL (Figure 1C, Table S2).

### 3.3. Functional Genes Involved in the C Cycle

**Figure 3:**
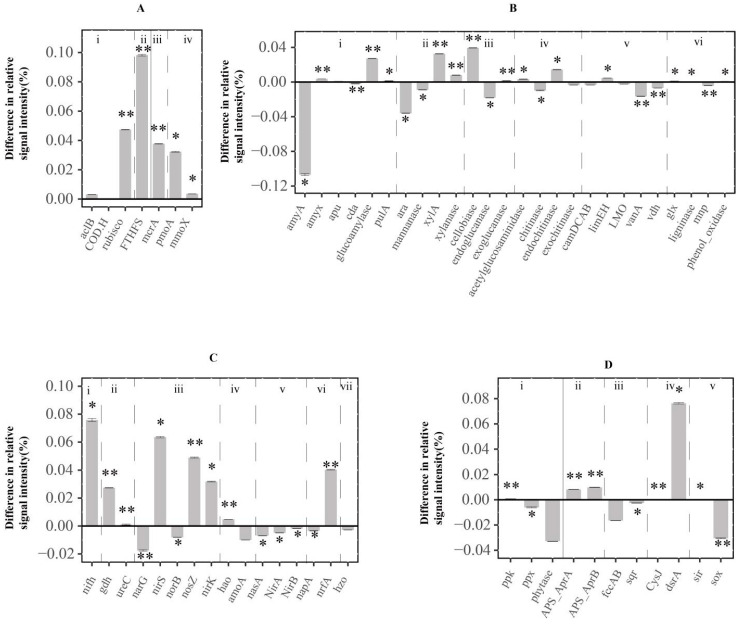
The differences in microbial metabolic potentials between the HL and the RAL based on the relative gene signal intensities. (**A**) Carbon fixation and methane cycling processes. i: autotrophic, ii: acetogenesis, iii: methanogenesis, iv: methane oxidation. (**B**) Carbon degradation processes. i: starch, ii: hemicellulose, iii: cellulose, iv: chitin, v: aromatic, vi: lignin. (**C**) Nitrogen cycling processes. i: nitrogen fixation, ii: ammonification iii: denitrification, iv: nitrification, v: assimilation N reduction, vi: dissimilation N reduction, vii: anammox. (**D**) Phosphorus cycling processes, sulfur cycling processes and metal metabolism. i: Phosphorus cycle and sulfur cycle (ii: adenylate sulfate reductase, iii: sulfide oxidation, iv: sulfite reduction, v: sulfur oxidation). The “*”and “**” indicate significant differences in gene intensities with *p* < 0.05 and *p* < 0.01, respectively, in the Student *t* test.

The carbon cycling gene category boasted the highest number of genes; the gene intensity from this category was notably higher in the HL than in the RAL (*p* < 0.05, Figure 3A,B).

For the carbon fixation pathways, the ATP citrate lyase (*aclB*), carbon-monoxide dehydrogenase (*CODH*), and ribulose-1,5-bisphosphate carboxylase/oxygenase (*rubisco*) gene groups were detected in both lakes, but only the rubisco gene (a key enzyme involved in the Calvin cycle [33]) showed a significant difference in signal intensity between the two lakes (*p* < 0.01, Figure 3A). The metabolic potential of acetogenesis (i.e., *FTHFS* (formyltetrahydrofolate synthetase) in the Wood–Ljungdahl pathway), methanogenesis (i.e., methyl coenzyme M reductase subunit A (*mcrA*)), methane oxidation (i.e., *pmoA* (particulate methane monooxygenase), and *mmoX* (soluable methane monooxygenase)) were also significantly higher in the HL (Figure 3A, *p* < 0.05 in all cases). Most of the carbon fixation genes were derived from Alphaproteobacteria and Gammaproteobacteria, and the relative proportions of these two bacteria were significantly higher in the HL (*p* < 0.05, Figure S1).

The total signal intensities of all the examined carbon degradation genes were higher in the HL compared with the RAL (Figure S2), which may be ascribed to the higher abundance of some common carbon degradation microorganisms, including Actinobacteria, Gammaproteobacteria, and Bacilli in the HL (*p* < 0.05, Figure S1). Compared with the RAL, the most significantly enhanced carbon degradation genes in the HL were related to cellulose metabolism (*cellobiase*, increasing 0.039%), two genes in hemicellulose metabolism (xylose isomerase (*xylA*) and xylanase, increasing 0.033% and 0.008%, respectively), and one involved in starch metabolism (glucoamylase, increasing 0.027%, Figure 3B). However, the relative signal intensities of two aromatic metabolizing genes (vanillate demethylase (*vanA*) and vanillin dehydrogenase *(vdh*)) and a lignin degradation gene (manganese peroxidase (*mnp*)) were lower in the HL (Figure 3B).

### 3.4. Functional Genes Involved in N Cycle

There were 16 gene groups affiliated in the N cycling category, among them the N fixation genes (nitrogenase gene (*nifH*)) were the most different between the HL and the RAL. The relative signal intensity of the *nifH* in the HL was 0.076% higher than that in the RAL (Figure 3C). The taxa–function relationship analysis revealed that the *nifH* is mainly derived from the Clostridia and Alphaproteobacteria, of which the relative abundances were significantly higher in the HL (*p* < 0.05, Figure S3). In addition, the two ammonification genes, glutamate dehydrogenase (*gdh*) and urease (*ureC*), increased 0.027% and 0.001%, respectively, more in the HL than in the RAL samples. The nitrification gene (hydroxylamine oxidoreductase (*hao*)) and the dissimilation N reduction gene (ammonia-forming cytochrome c nitrite reductase (*nrfA*)) increased 0.005% and 0.04% more in the HL than in the RAL samples, respectively (Figure 3C). The same patterns were also observed in the three denitrification genes (nitrous oxide reductase (*nosZ*), cytochrome cd1 nitrite reductas (*nirS*) and copper containing nitrite reductase (*nirK*), which increased 0.049%, 0.063%, and 0.032%, respectively (Figure 3C). Accordingly, the relative abundances of denitrifying bacteria in Alphaproteobacteria, Betaproteobacteria, and Gammaproteobacteria were significantly higher in HL samples (*p* < 0.05, Figure S3). By contrast, the metabolic potential of the assimilation N reduction in the RAL was higher than that in the HL (Figure 3C).

### 3.5. Functional Genes Involved in Phosphorus and Sulfur Cycle

Among the three phosphorus cycling genes, only the metabolic potential of polyphosphate biosynthesis (i.e., the *ppk* gene) was significantly higher in the HL than in the RAL (*p* < 0.01, Figure 3D). This was in accordance with the higher relative abundances of Gammaproteobacteria and Alphaproteobacteria in the HL (*p* < 0.05, Figure S4). By contrast, the phytase genes involved in phytic acid hydrolyzing and the *ppx* genes for inorganic polyphosphate degradation had significantly higher metabolic potentials in the RAL (*p* < 0.05, Figure 3D).

For the sulfur cycling gene category, the adenylate sulfate reductase genes and the sulfite reduction genes had significantly higher signal intensities in the HL (Figure 3D, *p* < 0.05, in all cases), which was in accordance with the higher abundance of microorganisms involved in adenylate sulfate reductase production (from Deltaproteobacteria and Gammaproteobacteria) and sulfite reduction (from Deltaproteobacteria) in the HL (Figure S4, *p* < 0.05, in all cases). However, the functional potential of a sulfur oxidation gene (*sox*) was significantly higher in the RAL compared with the HL (*p* < 0.01, Figure 3D).

### 3.6. Microbial Functional Gene Network Analyses

**Figure 4:**
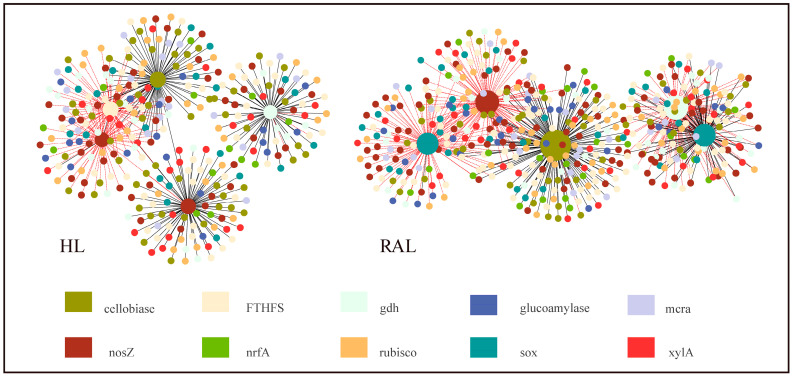
Differences in the network topology of key functional genes and the connectivity of the top five ranked genes in the HL and the RAL. The colors of the nodes indicate different functional genes. The solid black line indicates a positive relationship between the two nodes, and the short-dotted red line indicates a negative relationship. The size of each node is proportional to the number of connections (degrees).

Two functional gene molecular ecological networks were constructed, using 3275 genes in the HL and 2584 genes in the RAL samples, respectively. We obtained 2005 nodes and 5551 links (66.8% negative and 33.2% positive) for the HL, and 1703 nodes and 4667 links (54.5% negative and 45.5% positive) for the RAL networks, respectively (Figure 4).

The two networks were both scale-free (R^2^ > 0.85), and the clustering coefficients and harmonic geodesic distance were significantly different from those of the random networks (Table 2), suggesting that the fMENs of functional genes in both the HL and the RAL showed non-random small-world characteristics. Compared with the RAL network, the HL network generally had longer geodesic distances, lower clustering efficiencies, and more modules (Table 2). Two of the top five ranked genes in the HL were nosZ, and the other three were *FTHFS*, *cellobiase*, and *gdh*. Two of the top five ranked genes in the RAL were *sox*, and the other three were *cellobiase*, *nosZ*, and *gdh*. The network interactions of the five genes in the RAL were more complicated than those in the HL in terms of network connectivity and topology (Figure 4).

## 4. Discussion

### 4.1. Differences in Microbial Functional Gene Compositions and Structures between the HL and RAL

In this study, we investigated the functional gene compositions in microbial communities from an HL and a reference natural RAL and found higher diversity and lower homogeneity in the HL compared to the RAL samples (Figure 2). This may be attributed to the high content of DOC in the HL water due to an exceptionally large input of allochthonous organic matter [1,2]. As an important nutrient source that may regulate microbial communities [34], the higher DOC content might sustain more functional genes to show metabolic activity in HL samples (Figure 1A). Compared with the RAL, the HL had more special environmental conditions, e.g., low pH, poor light, low oxygen, but high content of HS (Table 1), which usually represents a harsh environment that filters out the adapted microbial species and functions [35], meaning lower homogeneity in the HL than that of the RAL (Figure 2).

For phylogenetic compositions, the HL and RAL shared the same dominant bacterial phyla, such as Proteobacteria, Actinobacteria, Bacteroidetes, Cyanobacteria, and Verrucomicrobia (Figure S5A), which was typical for most lake bacterioplankton communities [36]. However, at the class level, the two lakes had disparate phylogenetic compositions. Compared with the RAL, the relative abundances of the class Gammaproteobacteria, Bacteroidia, and Bacilli (especially the genus *Paenibacillus*) were higher in the HL (Figure S5B, Table S3). The persistent dominance of Gammaproteobacteria and Bacteroidia was also observed in another humic lake [9]. Interestingly, though the relative abundances of Alphaproteobacteria, Actinobacteria, and Verrucomicrobiae were lower, the relative abundances of some genus within these class, for example, *Rhodobacter* (Alphaproteobacteria), *Microbacteriaceae* (Alphaproteobacteria), and *Prosthecobacter* (Verrucomicrobiaewas) were much higher in the HL (Figure S5, Table S3). Some members of the *Rhodobacter* and *Prosthecobacter* had a high capacity for utilizing organic materials [37,38].

### 4.2. Differences in Metabolic Potentials between the HL and the RAL

Aquatic bacteria play key roles in greenhouse gas emissions in HLs due to their metabolic activities in utilizing the high content of organic carbon in water [39,40]. Genes for degradation of a wide range of carbon, ranging from the labile type (e.g., *glucoamylase*) to the recalcitrant type (e.g., *ligninase*), showed higher intensity in the HL than in the RAL, indicating that the high amount of carbon in the HL water could induce the “priming effect” for a variety of carbon types. Intriguingly, the carbon fixation gene (i.e., the *rubisco* gene) also showed a higher intensity in the HL samples, which may be ascribed to the fact that the HL inhabited more Gammaproteobacteria, from which many autotrophic microorganisms use the Calvin cycle for carbon fixation [41]. Previous studies have reported that HSs could stimulate CH_4_ oxidation by acting as an electron shuttle and extracellularly directing electrons to high-valent chemicals [42]. This stimulation of CH_4_ oxidation was verified by our study, in that the metabolic potential of methane oxidation (i.e., *pmoA* and *mmoX*) was significantly higher in the HL than in the RAL. Accordingly, the aerobic MOB from the genus *Methylobacter* (Gammaproteobacteria) were more abundant in the HL. The elevated methane oxidation may further lead to local oxygen scavenging [43], resulting in a positive-feedback loop to sustain higher potential methanogenesis (*mcr* gene more intensified, Figure 2) in anoxic microsites.

Our study showed that there was a significant difference in the relative signals of the *nifH* gene group between the HL and RAL (Figure 3C, *p* < 0.05). The main components of humic (humic acid and fulvic acid) can significantly increase the growth efficiency and nitrogen fixation capacity of N-fixing bacteria [44]. Denitrification, as a heterotrophic pathway, is an important link between the C and N cycles. Previous studies have revealed that humic acid could promote heterotrophic denitrifying bacteria, such as the *Thauera*, which could utilize HSs as an electron shuttle to improve denitrification performance, especially for nitrite reduction [45]. They were also detected in our study, and their *nosZ* and *nirS* intensities were significantly enhanced more in the HL than in the RAL (Student *t* test, both cases, *p* < 0.05). The relative intensities of most denitrification genes (except the *narG* and *norB*) were higher in the HL than in the RAL, implying that the denitrification processes (though not all steps) were promoted (Figure 3, *p* < 0.01), which may result in the quick removal of N in the water. This may help explain why there was much less difference in NO_X_^−^ contents, compared with the great difference in TN content, between the two lakes (Table 1). The enhanced nitrogen-fixing and denitrification processes implicated more rapid N cycling in the HL than in the clear RAL.

We observed enhanced microbial sulfate reduction potential but lower sulfide oxidation potential in the HL than in the RAL (Figure 3D). The high organic carbon availability in HLs was preferential for the heterotrophic sulfate-reducing bacteria [46], and the high content of humic substances often leads to lower oxygen levels in HL water [47], which may promote sulfur reduction but inhibit sulfur oxidation. The enhanced sulfate reduction and lower sulfide oxidation suggested more deposition of sulfur (e.g., in the form of sulfide metal) in the HL. We also observed less phosphorus degradation potential in the HL than in the RAL (Figure 3D). The humic acid fractions could inhibit phosphatase by binding with some active sites of plant-derived enzymes [48] and inhibit the activity of plant phytase by forming complexes with the enzyme substrates and having considerable absorption properties [49]. Our results suggest that similar mechanisms may also be responsible for microbial phosphatase and phytase (i.e., the lower *ppx* and *phytase* gene intensities) in the HL water.

### 4.3. Potential Interactions between Functional Genes

The special environmental characteristics of the HL resulted in distinct microbial community structures and also special functional gene network topological traits compared with the RAL. The negative links in the network may represent the competition or difference in metabolic preferences among community members [50,51]. In our study, the HL network had a higher proportion of negative correlations, which implicated that the refractory HSs aggravated the competition in HL microbial communities [35]. The networks with a shorter path length can transmit environmental fluctuations to the whole network in a shorter time and rapidly change the structure and function of the network [17]. The higher percentage of positive links in a network also favored quick and broad feedback in the community to environmental perturbations [52]. The average path distance was longer and the positive edge proportion was smaller in the HL, which indicated that the interactions between functional genes in the HL might be less efficient and respond less quickly to the changes in the environment than those in the RAL. Some studies have shown that the bacterial communities in a humic lake were relatively resilient to extreme weather events [53]. In addition, there was higher resource availability in the HL water (reflected by the higher contents of organic carbon, total nitrogen, phosphorus, and ferrum) (Table 1), which might reduce the inter-dependent relationships between different functional groups in acquiring nutrients for their growth [54]. Two of the top five high-degree genes in the HL network were the *nosZ* gene, highly connecting with the *cellobiase*, *nrfA*, and *xylA* genes, which reflected the enhanced nitrogen-reduction processes, and these processes depended on the degradation of organic carbons in the HL water. The highlighted *sox* gene in the RAL network reflected that the sulfur oxidation was characterized in the clear water of the RAL, which developed high correlations with the carbon-fixation genes (*rubisco* and *FTHFS*) and the *nosZ* gene. Similar gene co-occurrence of the *sox* and the carbon-fixation and denitrification genes were also found in a movile cave [55], which suggested that the chemolithoautotrophic sulfur-oxidizing bacteria might play a key role as a primary producer in the studied RAL ecosystem.

## 5. Conclusions

**Figure 5:**
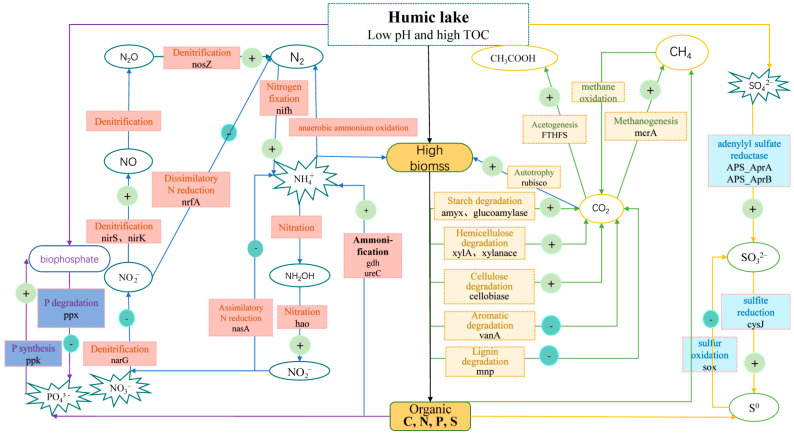
A conceptual model of the microbial metabolic processes in the HL. The “+” indicates that the microbes facilitate the process, and the “-” indicates that the microbes inhibit the process. Carbon, nitrogen, phosphorus, and sulfur flows are represented by green, blue, purple, and orange arrows, respectively. Biomass pools are shown as blue rectangular frames, and nutrients that could be directly assimilated by microbes are displayed by blue explosive graphics.

Our study showed that the special environmental characteristics in the HL water (high content of organic matter (with the humic substances as the main component), total nitrogen and phosphorus, and relatively low pH) could impact bacterioplankton communities to form unique functional gene compositions and result in disparate ecological processes compared with the reference RAL. The abundant organic matter might shed “priming effects” for the degradation of various carbon types in the HL and increase the metabolic potential of microorganisms to participate in processes such as methanogenesis, nitrogen fixation, and denitrification. These processes may lead to enhanced emissions of greenhouse gases (CO_2_, CH_4_, and N_2_O) in HLs [56]. However, the relatively low pH and some humic fractions in the HL water may exert inhibitions on the degradation of aromatic organic carbon, sulfur oxidation, and phosphorus degradation in the HL (Figure 5). The high amount of organic matter in the water might also change the interactions among different functional microorganisms, reducing the inter-dependent (or synergistic) relationships within the community to acquire nutrients for their growth. The validity of our results could be further tested using biogeochemical monitoring and other “omic” approaches (such as metatranscriptomics, metaproteomics, and metabolomics).

## Figures and Tables

**Table 1:** Physical and chemical properties for the HL and the RAL. pH, TOC, TP, TFe, TN, NO_x_^−^, conductivity, and area.

Lake	pH	TOC (mg·L^−1^)	TP (mg·L^−1^)	TFe (mg·L^−1^)	TN (mg·L^−1^)	NO_X_^−^ (mg·L^−1^)	Conductivity (μS·cm^−1^)	Area (km^2^)
HL	5.16	31.9	0.17	0.87	2.04	0.02	297	0.03
RAL	8.3	4.01	0.09	0.21	0.75	0	274	0.15

**Table 2:** Major topological properties of the empirical fMENs of core genes in the HL and the RAL and their associated random networks. * The number of genes used in the network construction. ^†^ The number of nodes in the fMEN. ^‡^ The parameters were generated from 100 randomly rewired networks.

Empirical Networks										Random Networks ^‡^		
Lake	No. of Original Genes *	Similarity Threshold (St)	Network Size (n) ^†^	No. of Links	R Square of Power-Law	Average Connectivity (avgK)	Harmonic Geodesic Distance (HD)	Average Clustering Coefficient (avgCC)	Modularity (No. of Modules)	Harmonic Geodesic Distance (HD ± SD)	Average Clustering Coefficient (avgCC ± SD)	Average Modularity (M ± SD)
HL	3275	0.99	2005	5551	0.88	5.537	4.96	0.21	0.842 (279)	3.668 ± 0.012	0.019 ± 0.002	0.396 ± 0.003
RAL	2584	0.99	1703	4667	0.852	5.481	4.585	0.297	0.834 (172)	3.401 ± 0.014	0.052 ± 0.003	0.396 ± 0.003

## Data Availability

The datasets generated during and/or analyzed during the current study are available from the corresponding author on reasonable request.

## Supplementary Materials

The following supporting information can be downloaded at: https://www.mdpi.com/article/10.3390/biology11101448/s1, Table S1: Full names of the enzyme/protein encoded by the functional gene groups; Table S2: Nonparametric multivariate statistical tests for MFS in HL and RAL; Table S3: The genus with 1% higher relative abundance of HL than RAL; Figure S1: Taxa–function relationships for carbon-cycling genes; Figure S2: The differences in the total intensities of certain biogeochemical cycling processes between HL and RAL, calculated by relative gene signal intensity; Figure S3: Taxa–function relationships for nitrogen-cycling genes; Figure S4: Taxa–function relationships for phosphorus and sulfur cycling genes; Figure S5: Taxa distributions in the HL and RAL (A: phylum, B: class) based on the 16S rRNA gene.
