# Supplementary material for: Humic Lake Exhibits Higher Microbial Functional Gene Diversity and Weaker Gene Interaction Efficiency than a Common Alkaline Lake
Source: Biology (Basel). 2022 Oct 1;11(10):1448. doi: 10.3390/biology11101448 (PMC9598760; doi:10.3390/biology11101448)

Supplementary materials

"Humic lake exhibits higher microbial functional gene diversity and weaker metabolic interaction efficiency than a common alkaline lake"

Dan He [1, 2,†], Yuanyuan Liu [3,†], Qinglong L. Wu [2, 4, 5], Yuyang Peng [3], and Lijuan Ren [2, 3,*]

[1] Guangdong Provincial Key Laboratory of Microbial Culture Collection and Application, State Key Laboratory of Applied Microbiology Southern China, Institute of Microbiology, Guangdong Academy of Sciences, Guangdong Detection Center of Microbiology, Guangzhou, China

[2] State Key Laboratory of Lake Science and Environment, Nanjing Institute of Geography and Limnology, Chinese Academy of Sciences, Nanjing, China

[3] Department of Ecology, Institute of Hydrobiology, Jinan University, Guangzhou, China

[4] Sino-Danish Centre for Education and Research, University of Chinese Academy of Sciences, Beijing, China

[5] Center for Evolution and Conservation Biology, Southern Marine Sciences and Engineering Guangdong Laboratory, Guangzhou, China

*Corresponding Author: Lijuan Ren

Corresponding Address:

Department of Ecology, Jinan University, Huangpu avenue 601#, Guangzhou, China.

E-mail: lijuanren@jnu.edu.cn

† The authors contributed equally to this work.

Short summary: this file contains 3 supplementary tables (Tables S1-S3) and 5 supplementary figures (Figures S1-S5)

Table S1. Full names of the enzyme/protein encoded by the functional gene groups.

Gene category	Subcategory	Gene name	Enzyme/protein encoded
Carbon fixation	Reductive tricarboxylic acid cycle	*aclB*	ATP citrate lyase
	Reductive acetyl–CoA pathway	*CODH*	Carbon monoxide dehydrogenase
	Wood–Ljungdahl pathway	*FTHFS*	Tetrahydrofolate formylase
	Calvin cycle	*rubisco*	RuBisCo
Methane	Methanogenesis	*mcrA*	Methyl coenzyme M reductase
	Methane oxidation	*mmoX*	Soluable methane monooxygenase
	Methane oxidation	*pmoA*	Particulate methane monooxygenase
Carbon degradation	Starch	*amyA*	Alpha-amylase
	Starch	*amyx*	Pullulanase
	Starch	*apu*	Amylopullulanase
	Starch	*cda*	Cyclomaltodextrinase
	Starch	*glucoamylase*	Glucoamylase
	Starch	*pulA*	Pullulanase
	Cellulose	*cellobiase*	Beta-glucosidase
	Cellulose	*endoglucanase*	Endoglucanase
	Cellulose	*exoglucanase*	Exoglucanase
	Hemicellulose	*ara*	Arabinofuranosidase
	Hemicellulose	*mannanase*	Beta-mannanase
	Hemicellulose	*xylA*	Xylose isomerase
	Hemicellulose	*xylanase*	Xylanase
	Chitin	*acetylglucosaminidase*	Acetylglucosaminidase

	Chitin	*chitinase*	chitinase
	Chitin	*endochitinase*	Endochitinase
	Chitin	*exochitinase*	Exochitinase
	Aromatics	*camDCAB*	Camphor 5-monooxygenase
	Aromatics	*limEH*	Limonene-1,2-epoxide hydrolase
	Aromatics	*LMO*	limonene monooxygenase
	Aromatics	*vanA*	Vanillate demethylase
	Aromatics	*vdh*	Aryl-aldehyde oxidase
	Lignin	*glx*	Glyoxal oxidase
	Lignin	*ligninase*	Lignin peroxidase
	Lignin	*mnp*	Manganese peroxidase
	Lignin	*phenol_oxidase*	Phenol oxidase
Nitrogen	Ammonification	*gdh*	Glutamate dehydrogenase
	Ammonification	*ureC*	Urease
	Anammox	*hzo*	Hydrazine oxidoreductase
	Assimilatory N reduction	*nasA*	Assimilatory nitrate reductase catalytic subunit
	Assimilatory N reduction	*nirA*	Nitrite reductase (NAD(P)H)
	Assimilatory N reduction	*nirB*	Nitrite reductase (NAD(P)H)
	Denitrification	*narG*	Respiratory nitrate reductase alpha chain
	Denitrification	*nirS*	Cytochrome cd1 nitrite reductas
	Denitrification	*norB*	Nitric oxide reductase
	Denitrification	*nosZ*	Nitrous oxide reductase
	Denitrification	*nirK*	Copper containing nitrite reductase
	Dissimilatory N reduction	*napA*	Periplasmic nitrate reductase
	Dissimilatory N reduction	*nrfA*	Ammonia-forming cytochrome c nitrite reductase

	Nitrification	*hao*	Hydroxylamine oxidoreductase
	Nitrification	*amoA*	Ammonia monooxygenase
	Nitrogen fixation	*nifH*	Nitrogenase iron protein
Phosphorus	Phytic acid hydrolysis	*phytase*	Phytase
	Polyphosphate synthesis	*ppk*	Polyphosphate kinase
	Polyphosphate degradation	*ppx*	Exopolyphosphatase
Sulfur	Adenylylsulfate reductase	*APS_AprA*	Adenylylsulfate reductase
	Adenylylsulfate reductase	*APS_AprB*	Adenylylsulfate reductase
	Sulfide oxidation	*fccAB*	Flavocytochrome c sulfide dehydrogenase
	Sulfide oxidation	*sqr*	Sulfide-quinone oxidoreductase
	Sulfite reductase	*CysJ*	Sulfite reductase (NADPH) flavoprotein subunit alpha
	Sulfite reductase	*dsrA*	Dissimilatory sulfite reductase
	Sulfite reductase	*sir*	Sulfite reductase (ferredoxin)
	Sulfur oxidation	*sox*	Sulfur oxidation cycle enzymes

Table S2. Nonparametric multivariate statistical tests for microbial functional structure in the HL and the RAL. Bolded numbers represent *P* < 0.01.

Index	MRPP		ANOSIM		ADONIS	
	δ	*P*	R	*P*	F	*P*
Bray–Curtis dissimilarity	0.147	**0.004**	0.520	**0.002**	5.598	**0.002**
Jaccard dissimilarity	0.215	**0.003**	0.546	**0.004**	5.575	**0.004**

Table S3. The clades with 1% higher relative abundance in the HL than in the RAL.

Genus	HL	RAL	Differences in relative abundance
Lelliottia	12.36%	0.00%	12.36%
Pseudomonas	8.27%	2.22%	6.05%
Edaphobaculum	4.84%	0.58%	4.25%
Comamonadaceae	3.71%	0.08%	3.63%
Sphingobacteriaceae	7.90%	4.36%	3.53%
Rhodobacter	2.61%	0.00%	2.61%
Microbacteriaceae	4.20%	1.62%	2.58%
Paenibacillus	2.49%	0.15%	2.34%
Sediminibacterium	2.45%	0.27%	2.18%
Methyloparacoccus	1.76%	0.00%	1.76%
Serratia	1.63%	0.46%	1.18%
Legionella	1.65%	0.54%	1.11%
Prosthecobacter	1.03%	0.04%	1.00%

Figure S1. Taxa–function relationships for carbon-cycling genes. Differences in relative signal intensity between a humic lake and a weakly alkaline lake were shown in the figure. i: carbon fixation, ii: methane, iii: common carbon degradation, iv: aromatic and lignin degradation.

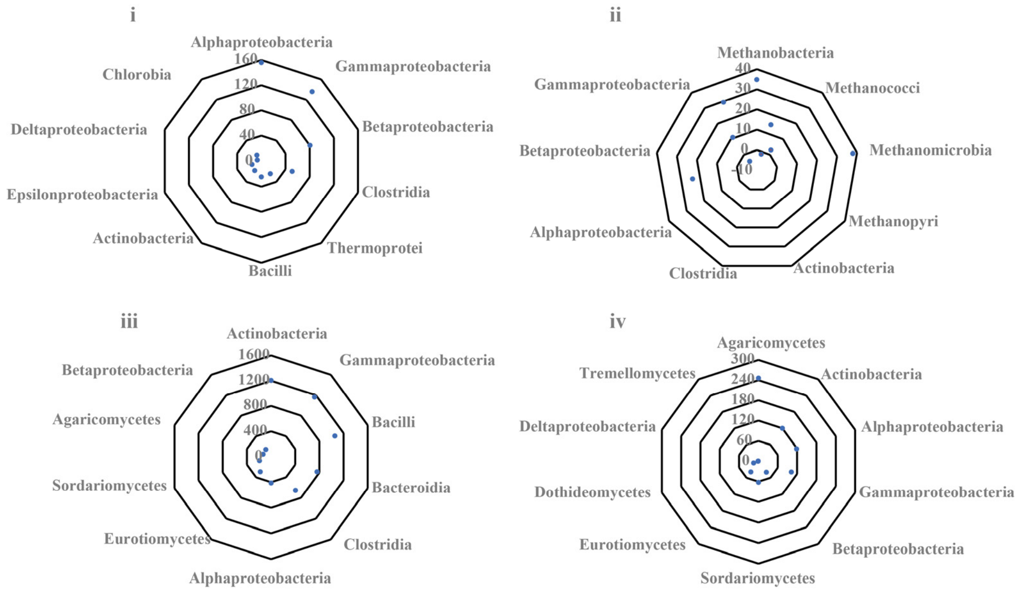

Figure S2. The differences in the total intensities of certain biogeochemical cycling processes between the HL and the RAL, calculated by relative gene signal intensity. A, carbon fixation process and methane cycling. i: autotrophic, ii: acetogenesis, iii: methanogenesis, iv: methane oxidation. B, carbon degradation processes. i: starch, ii: hemicellulose, iii: cellulose, iv: chitin, v: aromatic, vi: lignin. C, nitrogen cycle. i: nitrogen fixation, ii: ammonification iii: denitrification, iv: nitrification, v: assimilation N reduction, vi: dissimilation N reduction, vii: anammox. D, phosphorus cycle, sulfur cycle and metal metabolism. i: Phosphorus cycle, and sulfur cycle (ii: adenylate sulfate reductase, iii: sulfide oxidation, iv: sulfite reduction, v: sulfur oxidation). The “*”and “**” indicate significant differences in gene intensities with $P < 0.05$ and $P < 0.01$, respectively, in the student t test.

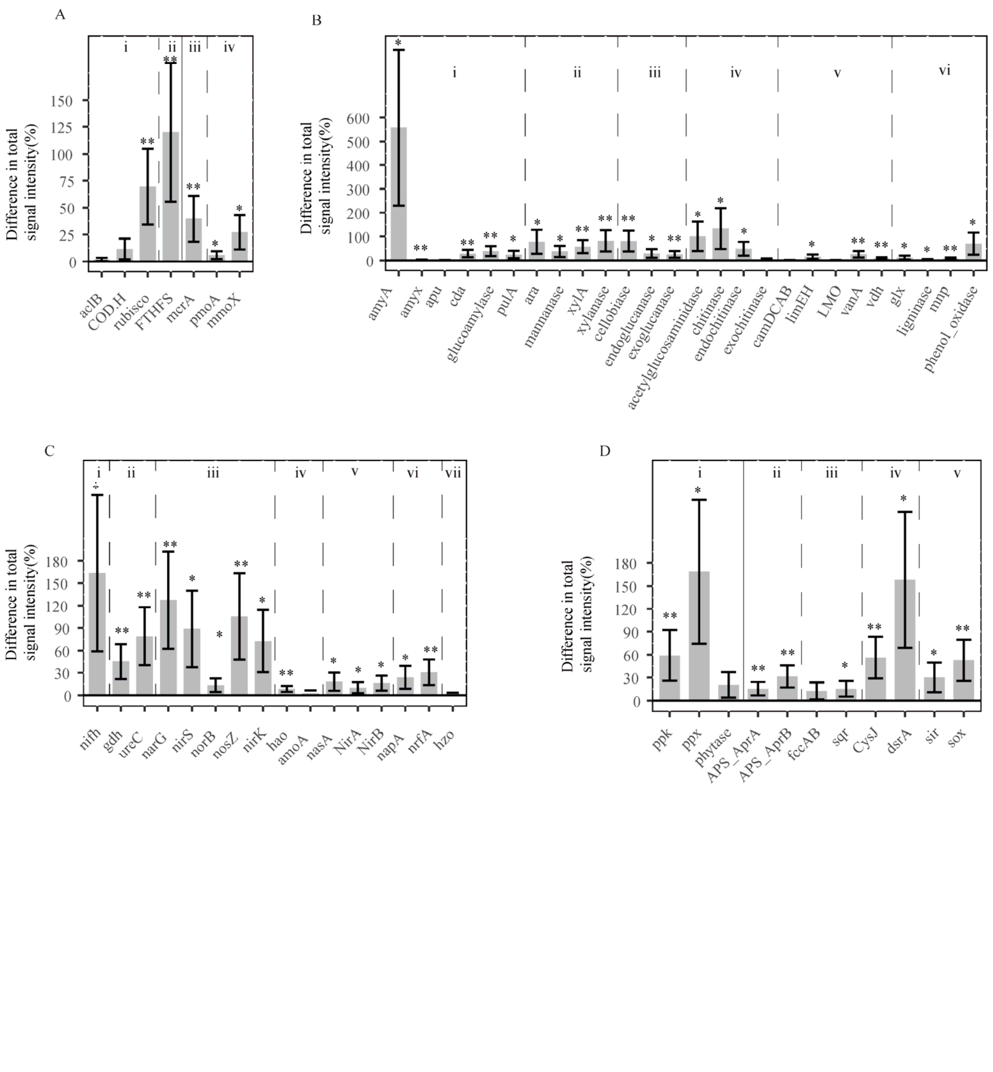

Figure S3. Taxa–function relationships for nitrogen-cycling genes. Differences in relative signal intensity between a humic lake and a weakly alkaline lake were shown in the figure. i: nitrogen fixation, ii: ammonification iii: denitrification, iv: nitrification, v: assimilation N reduction, and vi: dissimilation N reduction.

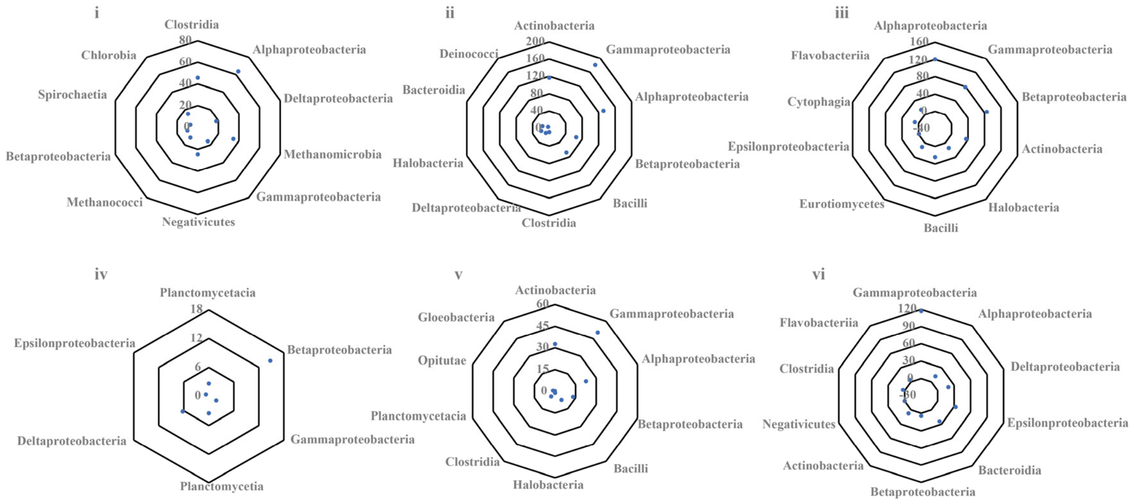

Figure S4. Taxa–function relationships for phosphorus and sulfur cycling genes. Differences in relative signal intensity between a humic lake and a weakly alkaline lake were shown in the figure. i: phosphorus cycle, ii: adenylate sulfate reductase, iii: sulfide oxidation, iv: sulfite reduction, v: sulfur oxidation.

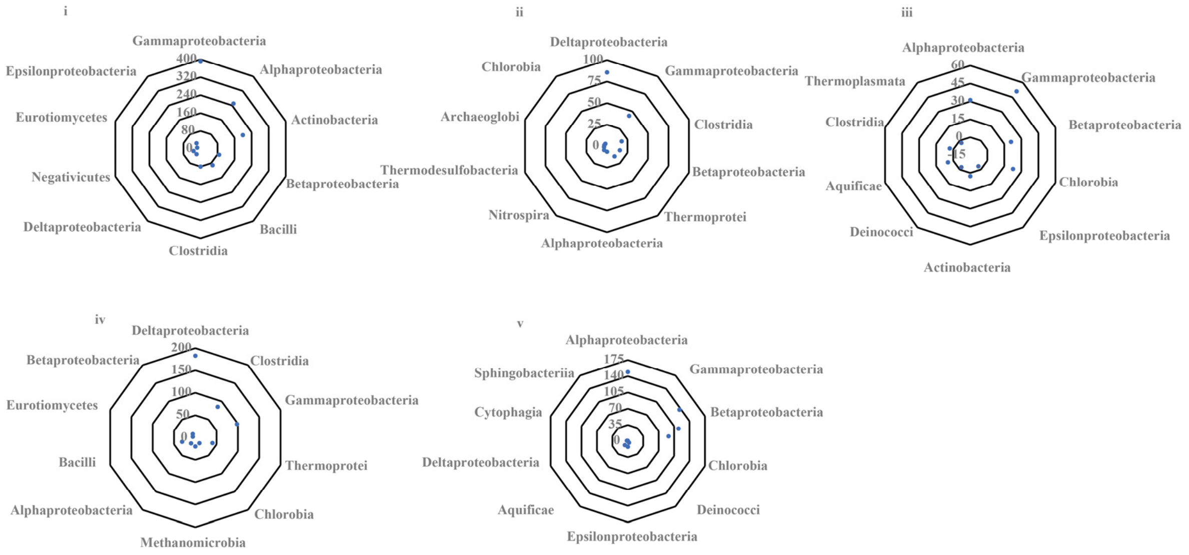

Figure S5. The distribution of microbial taxonomic compositions in the HL and the RAL (A: phylum, B: class).

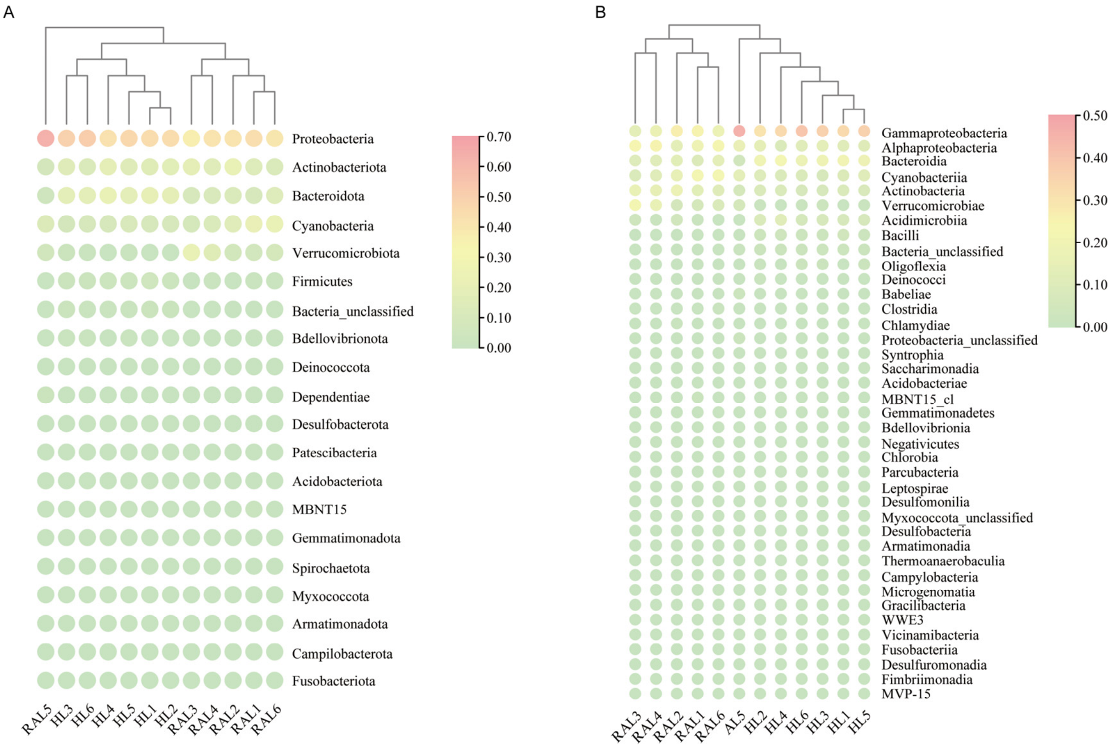

Supplement: Supplementary file 1 [file biology-11-01448-s001.zip › biology-1915760-supplementary.pdf]
